# Emergency Management of Post-Pancreatectomy Hemorrhage Secondary to a Ruptured Common Hepatic Artery Pseudoaneurysm: A Case Report

**DOI:** 10.5811/cpcem.39652

**Published:** 2025-05-28

**Authors:** Abigail Banks-McClelland, Terence Jackson, Nelson A. Royall

**Affiliations:** * Northeast Georgia Medical Center, Emergency Medicine Residency Program, Department of Graduate Medical Education, Gainesville, Georgia; † Northeast Georgia Medical Center Hepato-Pancreato-Biliary Surgery, Department of Surgery, Gainesville, Georgia; ‡ Northeast Georgia Medical Center, Hepato-Pancreato-Biliary Surgery, Department of Surgery, Department of Graduate Medical Education, Gainesville, Georgia

**Keywords:** pancreatoduodenectomy/Whipple procedure, post-pancreatectomy hemorrhage (PPH), sentinel bleed, postoperative pancreatic fistula (POPF)

## Abstract

**Introduction:**

Post-pancreatectomy hemorrhage (PPH) is a deadly complication of pancreatectomy procedures. Rapid identification of these life-threatening complications is crucial to mitigating associated morbidity and mortality rates. Delayed PPH is managed similarly to aortoenteric fistulas with damage control resuscitation and emergent endovascular interventions such as embolization and stent placement.

**Case Report:**

Here, we present the case of a delayed PPH presenting to the emergency department as a sentinel bleed secondary to a ruptured common hepatic artery pseudoaneurysm following a pancreatoduodenectomy.

**Conclusion:**

With hepatobiliary procedures being performed with more frequency, emergency physicians must be aware of the deadly postoperative complications such as post-pancreatectomy hemorrhage, their presentations, and their treatments.

## INTRODUCTION

Pancreatoduodenectomies are the primary treatment of periampullary masses and pancreatic malignancies, with a five-year overall survival rate of 50–70%.^1^ The pancreatoduodenectomy (also known as a Whipple procedure) removes the head and neck of the pancreas, duodenum (partial or complete), gallbladder, distal extrahepatic common bile duct, and associated lymphovascular tissues. The resection involves multiple vascular structures including the gastroduodenal and superior and inferior pancreaticoduodenal arteries. An anastomosis is created between the proximal jejunum and the body of the pancreas, proximal extrahepatic bile duct, and gastric antrum or proximal duodenum, with noted variations depending on surgeon preference (Figure).

Pancreatoduodenectomies, among other pancreatectomy procedures, have morbidity rates of up to 60% and mortality rates of up to 3% in high-volume centers.^2^ Postoperative pancreatic fistula (POPF) is a common postoperative complication in which pancreatic ductal fluid (and potentially enteric contents) leak from the pancreatojejunostomy anastomosis or pancreatic parenchyma, leading to profound local and systemic inflammation with occasional tissue necrosis.^1^–^6^ The POPF occurs in 10–40% of pancreatectomy procedures and has been identified in at least 50% of patients who develop delayed post-pancreatectomy hemorrhage (PPH).^1^ Post-pancreatectomy hemorrhage is believed to be due to pancreatic enzyme leakage creating ulceration of vascular walls or destruction of suture materials along ligated vascular structures such as the gastroduodenal or pancreaticoduodenal arteries.^1^–^6^ Although PPH occurs with an incidence of 3–4% of all pancreatectomies. it is responsible for 15–60% of postoperative mortalities in pancreatomy procedures.^3^–^5^

The International Study Group for Pancreatic Surgery has classified PPH based on timing in relation to the primary operation, location, and hemorrhage severity (Table 1). Early hemorrhage is within 24 hours of the procedure, while delayed hemorrhage is defined as any bleeding occurring more than 24 hours postoperatively.^3^ Hemorrhage can be intraluminal presenting as gastrointestinal (GI) bleeding, or extraluminal presenting as intra-abdominal bleeding.^3^ Mild PPH is defined as non-clinically significant bleeding that does not alter clinical course, while severe PPH is defined by the requirement of at least four units of packed red blood cell transfusion or a decrease in hemoglobin of at least 4 grams per deciliter.^3^ Grade A PPH occurs within 24 hours and does not change clinical course, while Grade C PPH is a late severe bleed that results in life-threatening hemorrhage and end organ damage (Table 1).^3^

*CPC-EM Capsule*

What do we already know about this clinical entity?

*Post-pancreatectomy hemorrhage may present with an intraabdominal or gastrointestinal sentinel bleed. These patients can quickly decompensate into profound hemorrhagic shock*.

What makes this presentation of disease reportable?

*This case highlights the importance of a high index of suspicion for and rapid identification of hemorrhagic complications in post-pancreatectomy patients, even those who are well-appearing*.

What is the major learning point?

*Post-pancreatectomy hemorrhage is ideally identified on early computized tomography angiography and should be treated using damage control resuscitation and emergency endovascular intervention*.

How might this improve emergency medicine practice?

*Greater awareness of post-pancreatectomy hemorrhage among emergency physicians may lead to more rapid identification and earlier implementation of life-saving intervention*.

Intra-abdominal PPH most frequently occurs along the gastroduodenal artery stump (16.7% of PPH), while bleeding from the common hepatic artery/proper hepatic artery not associated with other identified branches is less common (9% of PPH).^4^ Patients with delayed PPH have a significantly higher 90-day mortality rate when compared to patients without PPH (30.6% vs 6%, respectively).^4^ Half of delayed PPHs present with a sentinel bleed, which is a small bleed, either upper GI or intra-abdominal, that may herald an impending uncontrolled hemorrhage.^5^ The presence of a sentinel bleed alone is associated with a 57% mortality in pancreatectomy procedures.^5^ Here, we present the case of severe PPH secondary to a delayed ruptured common hepatic artery pseudoaneurysm following a pancreaticoduodenectomy complicated by POPF.

## CASE REPORT

This is the case of a 59-year-old male who presented to the emergency department (ED) nine days after an open pancreatoduodenectomy with distal gastrectomy and pancreaticojejunostomy. This patient’s immediate postoperative course was complicated by post-pancreatectomy pancreatitis and POPF with resultant delayed gastric emptying, which were significant predictors of delayed PPH. He had previously been diagnosed with a resectable, moderately differentiated pancreatic ductal adenocarcinoma of the head and completed 12 cycles of chemotherapy. He had undergone an endoscopic ultrasound with biopsy and endoscopic retrograde cholangiopancreatography (ERCP) with metal endobiliary stent placement complicated by post-ERCP pancreatitis prior to systemic therapy.

He was awoken the morning of the ninth postoperative day with severe exacerbation of epigastric abdominal pain despite home oral oxycodone. He presented emergently to the medical center where he was noted to have normal blood pressure and heart rate, but 75 milliliters of frank blood was noted in the abdominal drain. On abdominal exam, the patient did not present with any obvious peritonitis, only some epigastric discomfort. The lack of severe pain may have been secondary to the patient’s recent opioid treatment. The patient had adequate distal perfusion suggesting no active hemorrhage. Without signs of impending decompensation, the hepatobiliary service was consulted, and imaging was ordered while the patient received additional opioid medications to control the pain. Throughout the initial examination, he remained alert, oriented, and appropriate.

**Image 1:**
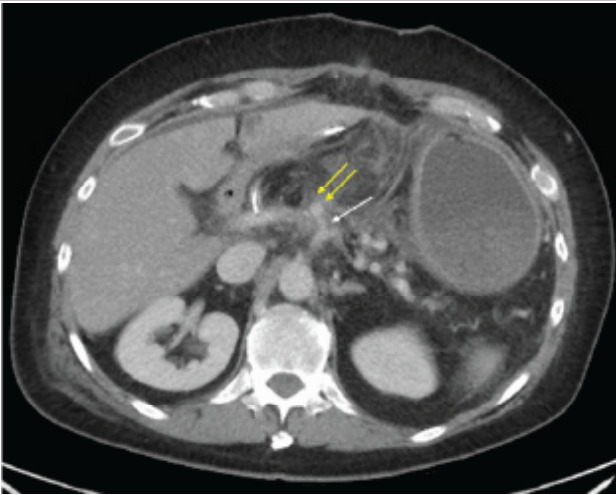
Axial computed tomography image with late arterial phase demonstrates a pseudoaneurysm (double yellow arrow) originating from the common hepatic artery (single white arrow). There is regional fat stranding related to pancreatoduodenectomy complicated by post-pancreatectomy pancreatitis.

Computed tomography (CT) of the abdomen and pelvis with intravenous (IV) contrast demonstrated a pseudoaneurysm along the common hepatic artery not adjacent to the gastroduodenal artery stump without active extravasation (Image 1). Laboratory workup revealed improving leukocytosis and hemoglobin from prior studies without other significant abnormalities.

During his evaluation in the ED, the patient developed sudden-onset tachycardia, hypotension, and altered mental status two hours after arrival. Repeat examination revealed severely decreased peripheral perfusion, pallor, and vital sign abnormalities suggestive of shock. At this time, the patient had rigidity and guarding in the abdomen, indicating peritonitis. Given the new accumulation of blood in the abdominal drain on presentation, shock was presumed to be secondary to hemorrhage. A second, large-bore peripheral IV access was obtained as the patient received two units of emergency release whole blood. He was initiated on norepinephrine with a goal systolic arterial pressure of 90 millimeters of mercury (mm Hg) in accordance with permissive hypotension. He was transferred emergently to the endovascular suite operating room for intervention.

**Image 2:**
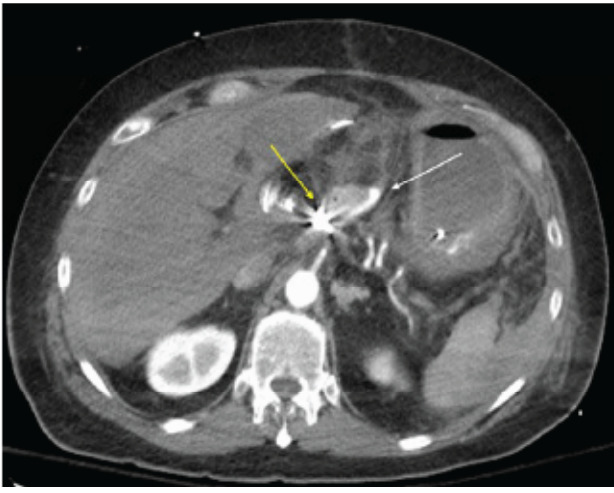
Axial computed tomography image in early arterial phase following angiography with endovascular coil embolization of the proximal common hepatic artery (single yellow arrow). There is noted absent arterial flow within the liver due to lack of replaced hepatic arterial flow. Previous extravasated contrast within the peritoneal cavity is shown (single white arrow) from the preceding angiography study.

During angiography he was found to have ruptured the common hepatic artery pseudoaneurysm with inability to place a covered metal endovascular stent or landing zone for embolization materials. Therefore, he underwent endovascular embolization of the common hepatic artery as a salvage therapy (Image 2). He was admitted to the surgical intensive care unit with ongoing post-hemorrhage resuscitation complicated by post-embolization acute liver failure, acute renal failure, acute respiratory failure, ventilator-associated pneumonia, pancreatic remnant necrosis, and atrial fibrillation. He unfortunately died seven weeks later following recurrent septic shock related to ischemic cholangitis.

## DISCUSSION

In this case, the patient presented to the ED with a sentinel extraluminal bleed identified by blood noted in the abdominal drain. He decompensated within hours of arrival when the common hepatic artery pseudoaneurysm ruptured. Follow-up imaging showed massive hemoperitoneum and angiography identified a likely source in the common hepatic artery. Unfortunately, the only means of hemorrhage control was to embolize the entire common hepatic artery. Due to rapidly increasing rates of pancreatic malignancies and improved rates of pancreatomy procedures being performed in the United States across academic and large community centers, it is essential for emergency physicians to be able to identify life-threatening complications of these procedures. This is not unlike the development of a secondary aortoenteric fistula (AEF) following aortic aneurysm repair, which frequently presents with a sentinel bleed in the form of GI bleeding. Severe delayed PPH is associated with sentinel bleeds 30–100% of the time.^3^,^7^ Sentinel bleeds can present as either GI bleeding (indicating likely intralumenal source) or increased drain output (indicating likely extraluminal source).^5^

Similar to the workup for a possible AEF, vascular imaging is required for diagnosis. While an AEF is best visualized with CT angiography of the abdomen, the imaging modality of choice in post-pancreatectomy patients is a CT pancreas protocol, which includes a non-contrast phase, an arterial phase, and a portal venous phase axial and coronal reconstructions to enable evaluation for pseudoaneurysms and portal venous thromboses or fluid collections.^7^,^8^ If vascular abnormalities or active bleeding is identified in delayed PPH, management involves emergent endovascular embolization/stenting (80% success rate).^5^ For patients exclusively diagnosed with early PPH, re-laparotomy is preferred with a 76% success rate.^5^ If a patient presents in hemorrhagic shock, he should undergo standard hemorrhagic shock interventions including whole blood transfusion, tranexamic acid, and thromboelastogram-based coagulopathy transfusion. The hemodynamic goals include permissive hypotension with a systolic pressure target of 90 mm Hg until vascular control has been achieved.^5^,^9^,^10^

Due to the frequent association of delayed gastric emptying with significant aspiration risk, early nasogastric decompression and endotracheal intubation should be considered. Emergent CT imaging if feasible is preferred, although in patients with persistent hemodynamic instability after initial transfusion upfront, angiography with intervention is required. Hemodynamic instability and end-organ damage associated with PPH indicates a grade C bleed, which has a mortality rate of 28.5%.^1^ Post-stabilization transfer of care to a high-volume pancreatectomy center is associated with improved survival for pancreatectomy patients due to significant risks for subsequent complications and failure to rescue.

## CONCLUSION

Post-pancreatectomy hemorrhage is a severe and life-threatening complication of pancreatic surgeries. Much like an aortoenteric fistula, delayed hemorrhage frequently presents with a sentinel bleed prior to massive hemorrhage with limited time frame for emergency endovascular interventions prior to hemodynamic collapse. Emergency physicians should be aware of the complications of pancreatic surgery and be prepared to promptly evaluate and mobilize resources. They should have a high suspicion of delayed PPH if a patient has had post-pancreatectomy pancreatitis, any documented post-operative pancreatic fistula or prolonged abdominal drainage symptoms of pancreatic or biliary juice leakage (worsening pain, delayed gastric emptying, or sentinel bleeding. Early CT pancreas protocol imaging and endovascular intervention to diagnose these patients can improve survival.

## Key Takeaways

- Post-pancreatectomy hemorrhage is a life-threatening complication of pancreatic surgery.
- Severe delayed PPH is the most common PPH sub-type presenting with a sentinel bleed.
- Treatment involves damage control resuscitation with blood products and emergent endovascular or surgical intervention.

## Figures and Tables

**Figure:**
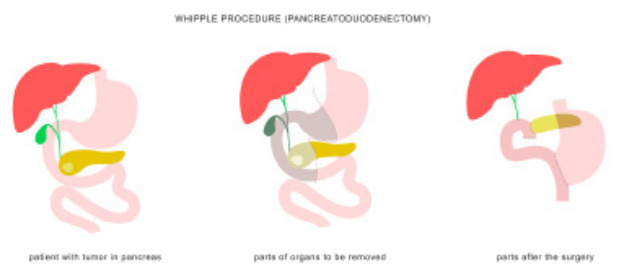
The pancreatoduodenectomy (Whipple procedure) involves the resection of the duodenum (partial or complete), pancreatic head and neck, extrahepatic bile duct (and gallbladder when present). The pancreatic neck, proximal extrahepatic bile duct, and proximal duodenum (or stomach) are anastomosed to the jejunum as shown. Medical infographic of Whipple procedure pancreaticoduodenectomy with gastrojejunostomy. Surgery operation in treatment of pancreatic cancer. By Aqua Art under Adobe standard license.

**Table 1:** Definition of post-pancreatectomy hemorrhage (PPH) and the classification of PPH as described by the International Study Group for Pancreatic Surgery.^3^

Definition of post-pancreatectomy homorrhage (PPH)			
Time of Onset	Location	Severity	
*Early:* within 24 hours of operation	*Intraluminal:* gastrointestinal source	*Mild:* small or medium volume blood loss (decrease in hemoglobin concentration less than 3 grams per deciliter (g/dL), mild clinical impairment without need for reoperation or interventional angiographic embolization)	
*Late:* more than 24 hours operation	*Extraluminal:* bleeding into abdominal cavity likely from vessels or resection area	*Severe:* large volume loss with drop in hemoglobin more than 3 g/dL, clinically significant impairment with vital sign changes, needs invasive treatment	
**Classification of PPH**			
**Grade**	**Time of onset, location, and severity**		**Clinical condition/intervention**
A	Early, intra- or extraluminal, mild		- Well -Observation and trending labs
B	Early, intra- or extraluminal, severe	Late, intra- or extraluminal mild to severe	-Well, rarely life-threatening -Observation with transfusions as needed, possible embolization
C		Late, intra- or extraluminal, severe	-Severely impaired, life-threatening -Angiography with localization of bleeding, embolization or possible open repair
